# Shading during the grain filling period increases 2-acetyl-1-pyrroline content in fragrant rice

**DOI:** 10.1186/s12284-015-0040-y

**Published:** 2015-02-10

**Authors:** Zhaowen Mo, Wu Li, Shenggang Pan, Timothy L Fitzgerald, Feng Xiao, Yongjian Tang, Yilei Wang, Meiyang Duan, Hua Tian, Xiangru Tang

**Affiliations:** College of Agriculture, South China Agricultural University, Guangzhou, Guangdong, 510642 China; Scientific Observing and Experimental Station of Crop Cultivation in South China, Ministry of Agriculture. P. R. China, Guangzhou, Guangdong 510642 China; Crops Research Institute, Guangdong Academy of Agricultural Sciences, Guangzhou, Guangdong 510640 China; CSIRO Agriculture Flagship, Queensland Bioscience Precinct, 306 Carmody Rd, St Lucia, Queensland 4067 Australia

**Keywords:** 2-acetyl-1-pyrroline, Aromatic rice, γ-aminobutyric acid, Proline, Shading treatment, Yield

## Abstract

**Background:**

Fragrant rice, including Thai jasmine and Indian basmati varieties, is highly valued by consumers globally. 2-acetyl-1-proline (2-AP) is the major compound responsible for the aromatic character of fragrant rice. Previously, environmental factors such as water management and salinity have been proven to influence 2-AP levels in fragrant rice; assessing the effect of additional environmental factors on 2-AP concentration is therefore eminent. The level of solar radiation (solar intensity; SI) to which a crop is exposed can affect growth, yield and grain quality, and other photosynthetic and physiological characteristics. In this study the effect of shading (i.e. the reduction of SI) on yield, quality, and 2-AP concentration in two elite Chinese fragrant rice varieties, ‘Yuxiangyouzhan’ and ‘Nongxiang 18’, has been investigated. Furthermore, accumulation of the plant stress response molecules proline and gamma-aminobutyric acid, which have also been implicated in pathways leading to 2-AP production, was assessed to study shading effects on these compounds in fragrant rice, and to further possibly determine fluxes in biochemical pathways leading to 2-AP accumulation.

**Results:**

This study has revealed significant changes in the yield and quality characters under shading treatment. Additionally, 2-AP and GABA content in grains was significantly increased for all shading treatments in both varieties. In addition to 2-AP, ten other volatile compounds were studied; results indicated that shading treatments could have a selective effect on the metabolism of these volatile compounds.

**Conclusions:**

In this study, we have demonstrated that shading during grain filling has significant effects on yield and quality traits in rice, and leads to the accumulation of GABA and 2-AP. We discuss the implications of these findings in terms of pathways leading to 2-AP and GABA production in fragrant rice, which have not been fully elucidated. The shading effect on ten additional volatile compounds is also discussed. Finally we discuss possible effects of variation in solar intensity resulting from anthropogenic emissions on fragrant rice production.

## Background

Fragrant rice varieties possess a characteristic aroma that is often described as ‘nutty’ or ‘popcorn-like’ (Bryant and McClung, 2010). Thai ‘jasmine’ and indian ‘basmati’ are the most widely-recognized types of fragrant rice. Fragrant rice is highly desired by consumers and attracts premium price in many markets (Sakthivel et al., 2009); furthermore, global demand for fragrant rice is increasing (Hashemi et al., 2013).

The volatile compounds in rice are complicated; instrumental analyses have observed more than 200 volatile compounds (Tsugita, 1985;Buttery et al., 1988; Champagne, 2008). Buttery et al. (1988) demonstrated that the probable key contributors to cooked rice aroma among the detected compounds were 2-acetyl-l-pyrroline, (E,E)-2,4-decadienal, nonanal, hexanal, (E)-2-nonenal, octanal, decanal, 4-vinyl-guaiacol, and 4-vinylphenol. Jezussek et al. (2002) suggested that 2-amino acetophenone and 4,5-epoxy-(E)-2-decenal were important previously unknown rice aroma compounds. Maraval et al. (2008) indicated that organic extracts of cooked aromatic rice showed similar aroma profile between the cultivars, but showed substantial differences in the levels of various volatile compounds. Moreover, Yang et al. (2008) suggested that 13 volatile coupounds may contribute to differences in aroma.

Despite the complexity of volatile compounds in rice (Champagne et al., 2005), a single volatile compound, 2-acetyl-1-pyrroline (2-AP), is well-proven to be primarily responsible for the aromatic character of fragrant rice (Buttery et al., 1983, 1986; Widjaja et al., 1996; Jezussek et al., 2002). In fragrant rice, 2-AP can be detected in all parts of the plant except the roots (Buttery et al., 1983; Maraval et al., 2010); 2-AP is also present in non-fragrant rice varieties, but at much lower concentration (Widjaja et al., 1996).

The inactivation of *BADH2*, encoding an aminoaldehyde dehydrogenase, is responsible for the accumulation of 2-AP in fragrant rice varieties (Bradbury et al., 2005; Bradbury et al., 2008). Proline is a precursor of 2-AP in rice (Yoshihashi et al., 2002), and γ-aminobutyraldehyde (GABald) is likely to be the direct precursor to 2-AP production via *BADH2*; Bradbury et al. (2008) proposed that functional *BADH2* catalyses the conversion of GABald to γ-aminobutyric acid (GABA), while non-functional *BADH2* in fragrant rice leads to cyclisation of GABald to Δ^1^-pyrroline, with the addition of an acetyl group forming 2-AP. The role of proline in plant stress tolerance is well-established (Szabados and Savouré, 2010); additionally, GABA accumulates in response to biotic and abiotic stresses in plants (Bouche and Fromm, 2004) and is therefore speculated to play a role in stress tolerance (Kinnersley and Turano, 2000). Thus, it appears that there is an overlap in pathways involved in 2-AP accumulation and the response to stress. In further support of an interaction between 2-AP production and stress responses, environmental factors have been observed to influence the level of 2-AP in fragrant rice grains. For example, Yoshihashi et al. (2002) reported that drought stress during grain formation increased 2-AP content, and Gay et al. (2010) and Poonlaphdecha et al. (2012) have also recently reported an increase in 2-AP accumulation in response to salt stress. Interestingly, elite fragrant rice varieties are relatively susceptible to abiotic and biotic stresses (Niu et al., 2008), and recently an association of the fragrance phenotype with salt susceptibility has also been reported (Fitzgerald et al., 2010; Wijerathna et al., 2014).

‘Photosynthetically Active Radiation’ (PAR) is the range of electromagnetic radiation between 400 and 700 nanometers, roughly equivalent to the visible light spectrum, that plants (and most photosynthetic organisms) harness for photosynthesis. The level of PAR to which a plant is exposed is related to photosynthetic rate (McCree, 1981), and variation in PAR can therefore have a major effect on plant growth and development. In the field, the intensity of solar radiation (solar intensity; SI) determines the level of PAR to which a crop is exposed, and variation in solar radiation can affect crop yield (Liu and Tollenaar, 2009). Season, geographic location, and cloud cover are key factors influencing SI; additionally, SI varies in response to atmospheric pollution and water vapour content (Jáuregui and Luyando, 1999; Haywood et al., 2011). The level of SI to which a plant is exposed can also be strongly affected by surrounding vegetation; ‘shading stress’ in plants growing beneath or amongst a canopy, and responses to such stress, are well-recognised phenomena (Gommers et al., 2013).

To understand how rice responds to low light environment, researchers have used artificial shading to control light density. Studies have showed that shading can affect rice morphological characteristics, physiological characteristics, yield, and quality. For example, Tang (1988) found that shading affected growth and yield, decreased dry matter, and increased leaf thickness. Cai and Luo (1999) reported that shading at different stages decreased rice yield, accumulation rate of dry matter and the uptake of nutrients, but increased nutrient content. Ren et al. (2003) indicated that the distribution and transformation of plant dry matter is affected by shading, and some shading levels decreased rice yield. Besides, Deng et al. (2009) found that the filled grain percentage and yield were obviously affected by shading. Moreover, Liu et al. (2012) reported that the effect of shading on the content of malondialdehyde, souble sugar, souble protein, and protective enzymes activities in leaves various in genotype. Ding et al. (2004) found that shading after heading extended rice growth duration of the flag leaf, decreased decomposing rate of chlorophyll and MDA content, reduced the photosynthesis of the flag leaf, and that the degree of the grain-filling was lower than that of under the strong light. Zhang et al. (2007) indicated that shading affected rice quality differently amongst genotypes, however shading increased protein content and chalkiness and decreased amylose content of all rice cultivars. Liu et al. (2008) found that weak light during grain filling affected rice yield, physiological characteristics and quality, particularly the rate of chalky grains. Additionally, shading has been demonstrated to effect starch synthase and related enzyme activities (Wang et al., 2013).

Previous studies have assessed the effects of an artificial shading (which results in a decrease in SI and PAR) on growth and development, yield, and grain quality in rice (e.g. Tanaka and Kawano, 1966; Cai and Luo, 1999; Ren et al., 2003; Zhang et al., 2007). Here we have studied the impact of shading during the grain filling period on GABA, proline, and 2-AP accumulation in fragrant rice. Furthermore, to provide additional insight into the effect of shading on growth and yield in fragrant rice, we have complemented our analyses of metabolite accumulation with assessment of total nitrogen levels, and grain yield and quality in response to shading treatment.

## Results

### Effect of shading treatment on yield, yield related traits, dry weight, and harvest index

For Yuxiangyouzhan, all shading treatments resulted in a significant reduction in filled grain percentage, 1000-grain weight, grain yield, total dry weight, and harvest index. Additionally, shading treatment during the whole grain filling period (S1) and during the latter stage of grain filling (S3) caused a significant reduction of panicle number. For Nongxiang 18, shading treatment during the whole grain filling stage S1 and during early grain filling (S2) caused a significant reduction of filled grain percentage and 1000-grain weight; and S1 significantly decreased grain yield and total dry weight (Table 1). On average, Yuxiangyouzhan had lower panicles number, filled grain percentage, 1000-grain weight, and total dry weight, but had a higher number of grains per panicle, grain yield, and harvest index than Nongxiang 18 (Table 1).

**Table 1:** **Effect of shading treatment on yield, yield related traits, total dry weight, and harvest index**

**Treatment**		**Panicles number (m** ^**2**^ **)**	**Grains per panicle**	**Filled grain percentage (%)**	**1000-grain weight (g)**	**Grain yield (g**_∙_ **m** ^**−2**^ **)**	**Total dry weight(g**_∙_ **m** ^**−2**^ **)**	**Harvest index (%)**
Yuxiangyouzhan								
	S0	263.33 a	185.09 a	84.48 a	21.27 a	863.14 a	1611.0 a	53.613 a
	S1	190.00 c	162.63 a	51.61 c	19.51 b	494.04 c	1021.0 c	48.311 b
	S2	250.00 ab	165.47 a	63.38 b	19.58 b	660.66 b	1328.5 b	49.602 b
	S3	218.33 bc	179.61 a	64.74 b	20.04 b	523.69 c	1100.4 c	47.512 b
	mean	230.42	173.20*	66.05	20.10	635.38	1265.2	49.759*
Nongxiang 18								
	S0	284.42 a	148.41 a	85.61 a	25.46 a	655.19 a	1453.8 a	44.783 a
	S1	261.67 a	126.64 a	53.32 c	22.94 b	457.25 b	1100.4 b	41.495 a
	S2	290.00 a	129.09 a	66.01 b	23.07 b	639.96 a	1394.9 a	45.841 a
	S3	273.33 a	148.32 a	81.76 a	24.84 a	638.35 a	1376.3 a	46.084 a
	mean	277.35*	138.11	71.68	24.08*	597.68	1331.4	44.551

Means in the same column followed by different lower case letters for the same variety differ significantly at *P* = 0.05 by LSD tests. Means of the two varieties followed by asterisk for the same detected index difer significant at *P* = 0.05 by LSD tests.

### Effect of shading treatment on grain quality

Significant effects were identified for some shading treatments on most quality traits. All shading treatments significantly increased grain protein content in both Yuxiangyouzhan and Nongxiang 18 (Table 2). Additionally, a similar effect of shading on chalkiness characterstics was observed in both cultivars; shading during the whole grain filling period and during late grain filling resulted in a decrease in mean rate of chalky grains, and mean degree of chalkiness, however shading during early grain filling significantly increased chalkiness characteristics (Table 2). No significant effect on brown rice rate was detected in the shading treatments for either cultivar, while for milled rice rate, head rice rate and alkali spreading value effects varied with timing, during shading as well as cultivar (Table 2).

**Table 2:** **Effect of shading treatment on grain quality**

**Treatment**		**Brown rice rate (%)**	**Milled rice rate (%)**	**Head rice rate(%)**	**Protein content(%)**	**Amylose content(%)**	**Alkali**	**grains with chalkiness (%)**	**Chalkiness degree (%)**
Yuxiangyouzhan									
	S0	83.81 a	73.00 ab	70.25 a	9.53 c	23.43 c	7.47 ab	23.33 b	8.63 b
	S1	83.56 a	72.66 bc	69.99 a	10.23 b	23.67 c	7.37 b	14.33 c	7.33 b
	S2	83.62 a	72.29 c	68.29 b	10.40 a	24.90 b	7.57 a	32.00 a	18.17 a
	S3	83.71 a	73.14 a	70.48 a	9.23 d	25.97 a	7.40 ab	18.67 bc	7.70 b
	mean	83.67*	72.77*	69.75*	9.85*	24.49*	7.45*	22.08*	10.46*
Nongxiang 18									
	S0	81.97 a	68.40 b	64.43 b	8.43 d	19.20 a	6.60 a	4.33 b	1.30 b
	S1	82.43 a	69.21 ab	64.29 b	9.17 a	18.23 a	6.60 a	2.33 c	0.71 c
	S2	81.64 a	68.58 b	64.57 b	8.97 b	18.50 a	6.63 a	13.67 a	3.00 a
	S3	82.52 a	69.77 a	66.39 a	8.60 c	18.50 a	6.53 a	2.33 c	0.73 c
	mean	82.14	68.99	64.92	8.79	18.61	6.59	5.67	1.44

Means in the same column followed by different lower case letters for the same variety differ significantly at *P* = 0.05 by LSD tests. Means of the two varieties followed by asterisk for the same grain quality trait difer significant at *P* = 0.05 by LSD tests.

### Effect of shading treatment on 2-acetyl-1-pyrroline, GABA, proline, and total nitrogen levels

**Figure 1:**
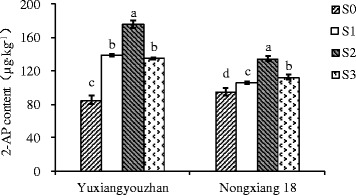
**Effect of shading treatment on grain 2-AP content in grains.** Vertical bars with different lower case letters above are significantly different at *P* = 0.05 by LSD tests. Capped bars represent SD.

**Figure 2:**
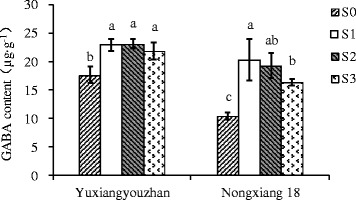
**Effect of shading treatment on grain GABA content in grains.** Vertical bars with different lower case letters above are significantly different at *P* = 0.05 by LSD tests. Capped bars represent SD.

**Figure 3:**
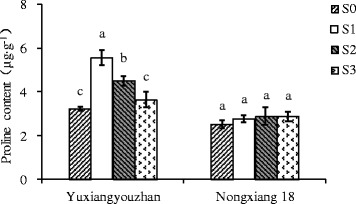
**Effect of shading treatment on proline content in grains.** Vertical bars with different lower case letters above are significantly different at *P* = 0.05 by LSD tests. Capped bars represent SD.

**Figure 4:**
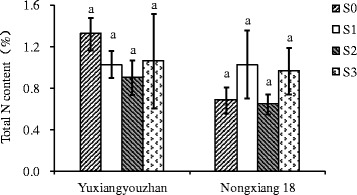
**Effect of shading treatment on total nitrogen content in grains.** Vertical bars with different lower case letters above are significantly different at *P* = 0.05 by LSD tests. Capped bars represent SD.

The 2-AP content in grains was significantly increased for all shading treatments in both varieties, with increases of 59.07 – 106.65%, and 11.89 – 42.37% detected for Yuxiangyouzhan and Nongxiang 18, respectively. In both varieties, the highest mean 2-AP content in grains was identified for plants exposed to S2 treatment, with 2-AP contents of 175.86 μg∙kg^−1^ in Yuxiangyouzhan and 135.02 μg∙kg^−1^ in Nongxiang 18, compared to respective means of 85.10 and 94.84 μg∙kg^−1^ in the absence of shading (S0) (Figure 1). All shading treatments also significantly increased GABA in the grains of Yuxiangyouzhan (23.59 - 31.01% ) and Nongxiang 18 (56.99 - 94.18%) (Figure 2). In Yuxiangyouzhan, a significant increase in proline content was identified for S1 and S2, but not for S3 (Figure 3). For Nongxiang 18, proline content was not significantly affected by any shading treatment (Figure 3). No significant difference in total nitrogen content in grains between shading treatments was observed (Figure 4).

### Relationships among 2-Acetyl-1-pyrroline, GABA, proline, and total nitrogen

Significant correlations were identified between 2-AP and GABA in response to shading for Yuxiangyouzhan but none for Nongxiang 18. No correlation was found between total nitrogen and GABA, proline or 2-AP and between proline and total nitrogen, GABA or 2-AP in either variety (Table 3).

**Table 3:** **Correlation coefficients among 2-AP, GABA, total nitrogen, and proline content in grains**

**Index**	**2-AP content**	**Grain GABA content**	**Proline content**	**Total nitrogen content**
Yuxiangyouzhan				
2-AP content	1	0.8326**	0.5460	−0.5557
GABA content	0.8326**	1	0.6926	−0.3402
Proline content	0.5460	0.6926	1	−0.2272
Total nitrogen content	−0.5557	−0.3402	−0.2272	1
Nongxiang 18				
2-AP content	1	0.5442	0.5356	−0.1758
GABA content	0.5442	1	0.3565	0.3836
Proline content	0.5356	0.3565	1	0.3267
Total nitrogen content	−0.1758	0.3836	0.3267	1

Significant correlations at **p < 0.01.

**Table 4:** **Effect of shading treatment on the relative content (%) volatile compounds in grains**

**Compounds**	**Yuxiangyouzhan**					**Nongxiang 18**				
	**S0**	**S1**	**S2**	**S3**	**Mean**	**S0**	**S1**	**S2**	**S3**	**Mean**
(E)-2-Hexenal	0.58c	1.76a	1.67a	1.17b	1.29	1.15b	2.16a	1.86a	1.55ab	1.68*
1-Hexanol	1.45a	1.12a	1.65a	1.69a	1.48	2.10ab	2.55a	2.21ab	1.78b	2.16
Heptanal	15.73a	19.84a	17.97a	16.28a	17.45	25.11ab	22.99b	24.14ab	25.91a	24.51*
2-acetyl-1-pyrroline	18.06b	18.07b	18.08b	22.93a	19.29*	10.84b	14.22a	14.17a	13.11a	13.08
Octane	7.26a	7.22a	5.91b	5.83b	6.55	7.00ab	7.47a	6.25bc	6.01c	6.68*
1-Heptanol	1.29a	1.07a	0.85a	0.86a	1.02	1.06a	0.98a	1.29a	1.02a	1.09
1-Octen-3-ol	1.96a	2.16a	3.43a	1.80a	2.34	2.12a	1.94a	1.87a	2.08a	2.00
Octanal	11.92b	14.81a	13.56ab	11.60b	12.97	13.36b	13.62b	13.26b	15.24a	13.87*
Benzyl alcohol	0.24b	0.74a	0.38ab	0.61ab	0.52	0.20b	0.78a	0.27b	0.82a	0.49
Benzeneacetaldehyde	5.31a	3.95a	4.00a	5.53a	4.70	4.16ab	4.90a	4.16ab	3.73b	4.23
3,8-Dimethylundecane	12.08a	9.62a	9.81a	13.36a	11.22	9.46b	12.16a	12.22a	8.67b	10.63

Means in the same row followed by different lower case letters for the same variety differ significantly at *P* = 0.05 by LSD tests. Means of the two varieties followed by asterisk for the same compound difer significant at *P* = 0.05 by LSD tests.

### Effect of shading treatment on the relative content (%) volatile compounds

Eleven volatile compounds were measured: (E)-2-Hexenal, 1-Hexanol, Heptanal, 2-acetyl-1-pyrroline, Octane, 1-Heptanol,1-Octen-3-ol, Octanal, Benzyl alcohol, Benzeneacetaldehyde, and 3,8-Dimethylundecane. For Yuxiangyouzhan, shading treatments significantly increased relative content in (E)-2-Hexenal. S3 significantly increased relative content in 2-AP. Shading treatments (S2 and S3) significantly decreased relative content in Octane. S1 treatment significantly increased the relative content of Octanal and Benzyl alcohol. For Nongxiang 18, shading treatments (S2 and S3) significantly increased the relative content of (E)-2-Hexenal. Shading treatments significantly increased the relative content of 2-AP. S3 significantly decreased the relative content of Octane, while significantly increased the relative content of Octanal. Shading treatment (S1 and S3) significantly increased the relative content of Benzyl alcohol. Shading treatments (S1and S2) significantly increased the relative content of 3,8-Dimethylundecane. As for the comparision between the two varieties, Yuxiangyouzhan had a significant higher relative content of 2-AP than Nongxiang 18. The relative content of (E)-2-Hexenal, Heptanal, Octane,and Octanal in Nongxiang 18 was significantly higher than that of Yuxiangyouzhan (Table 4).

## Discussion

Shading limits photosynthetically active radiation (PAR) and can be a source of plant stress. Several studies have assessed the effect of shading on growth and yield in cereal crops including rice; shading during flowering and grain development has been consistently found to decrease yield (e.g. Beed et al., 2007; Zhang et al., 2006; Liu and Tollenaar, 2009; Deng et al., 2009). Zhang et al. (2007) found that shading affects quality traits in rice, and generally, shading resulted in increased grain protein content and decreased amylose content, as well as an increased rate of chalky rice grains and a greater average degree of chalkinesss. In this study, during the whole (S1), early (S2), or late (S3) grain filling period, shading treatment resulted in significantly decreased yield in two elite Chinese fragrant rice varieties, with S1 treatment resulting in the greatest yield losses. Yield losses were more substantial in Yuxiangyouzhan than Nongxiang 18 under all shading treatments; this is consistent with previous studies in which genotype has been found to influence yield loss due to shading in rice (Zhu et al., 2008; Deng et al., 2009). Shading treatment also significantly affected quality traits. An increase in grain protein content under all treatments and a consistent effect of treatment on grain chalkiness were observed for both varieties. Significant shading effects were also identified for most other quality traits assessed, however these varied with timing, duration of treatment, and cultivar. Together, these results are largely consistent with previous literature and confirm that shading during grain development can have a substantial effect on grain yield and quality in fragrant rice.

Here we have found that all shading treatments significantly increased the concentration of both 2-AP and GABA in the grain of Yuxiangyouzhan than Nongxiang 18; furthermore, positive correlation between the accumulation of these compounds was observed in both varieties. Although GABA has been shown to accumulate in response to a range of stresses, direct assessment of the impact of shade on GABA accumulation appears limited; our results demonstrate that the conserved response of GABA to plant stress extends to shading in rice. Furthermore, our results indicate that the association between plant stress and accumulation of 2-AP exists for shading in addition to drought and salt. We also assessed accumulation of proline in reponse to shading treatment. This was significantly increased in Yuxiangyouzhan by S1 and S2 treatment; in contrast, no significant effect was detected for any shading treatment in Nongxiang 18. This suggests that there may be substantial variation of proline accumulation in response to shading in fragrant rice varieties.

Our observations of the accumulation of GABA and 2-AP in response to shading draw attention to the hypothesized relationship of the biochemistry of these compounds in fragrant rice. As outlined above, *in vitro* rice BADH2 catalyses the conversion of GABald to GABA, a molecule that has been widely implicated in plant stress responses (Bouche and Fromm, 2004; Kinnersley and Turano, 2000) and it has been hypothesized that in fragrant rice non-functional *BADH2* leads to the accumulation of GABald, which cyclises and reacts with acetyl group forming 2-AP (Bradbury et al., 2008). However, although GABA can be synthesized in plants via GABald, the primary pathway for GABA production is thought to be the ‘GABA shunt’, in which glutamate is converted to GABA via glutamate decarboxylase (GAD) (Fait et al., 2008).

If 2-AP indeed accumulates in fragrant rice via a pathway that produces GABA from GABald in the presence of functional *BADH2* (Bradbury et al., 2008), it is intriguing that the accumulation of both GABA and 2-AP increases in response to shade. This suggests that there may be parallel activation of pathways leading to GABA in response to shading in fragrant rice, with 2-AP accumulating from GABald rather than GABA due to non-functional *BADH2*, but GABA also accumulating via the GABA shunt. However, it is possible that the accumulation of both GABA and 2-AP occurs via GABald. If another enzyme exists in rice that catalyses the conversion of GABald to GABA, non-functional BADH2 may result in a loss of efficiency rather than elimination of GABA accumulation via GABald, with excess GABald leading to accumulation of 2-AP. Bradbury et al., (2008) demonstrated that BADH1, an enzyme with ~ 76% identity to BADH2, catalyses the formation of GABA from GABald *in vitro*, albeit at lower efficiency than BADH2. Furthermore, Singh et al., (2010) identified an association of SNPs predicted to decrease activity of BADH1 on GABald with 2-AP-based aroma strength in rice. Thus, this latter possibility seems plausible. In either case, modulation of GABA accumulation in response to stress seems a credible explanation for the decrease in stress tolerance reported to be associated with fragrance in rice (Fitzgerald et al., 2010; Wijerathna et al., 2014).

For the other volatile compounds in grains, Buttery et al. (1988) demonstrated ten probable key contributors to cooked rice aroma among the detected compounds. Two of the ten volatile compounds, 2-acetyl-l-pyrroline and Octanal have been studied extensively (e.g. Mahatheeranont et al., 2001; Jezussek et al., 2002; Wongpornchai et al., 2003; Laohakunjit and Noomhorm, 2004; Yang et al., 2008; Maraval et al., 2008; Tananuwong and Lertsiri, 2010; Goufo et al., 2011; Goufo et al., 2010; Bryant and McClung, 2010; Mathure et al., 2014; Mahattanatawee and Rouseff, 2014; Liyanaarachchi et al., 2014). In our study, 2-AP had the highest average relative content among the detected compounds in Yuxiangyouzhan, while was the the third most abundant volatile detected in Nongxiang 18. Octanal had the second higher average relative content among the detected compounds in both varieties. There was a significant difference in the average average relative content of the two compounds between the two varieties. All shading treatment significantly increased the relative content in 2-AP in Nongxiang 18, while S3 had a significant impact on relative content in 2-AP in Yuxiangyouzhan. S1 significantly increased the relative content of octanal in both varieties. Of the compounds that were posited to contribute to fragrance in rice by Yang et al. (2008), in addition to 2-AP and octanal, heptanal and 1-octen-3-ol were also detected in our studies; and these two compounds have also been reported in other studies (Yang et al., 2008; Tananuwong and Lertsiri, 2010; Goufo et al., 2010; Bryant and McClung, 2010; Mathure et al., 2014). We found that, heptanal had the highest average relative content in Nongxiang 18, and had the second high average relative content in Yuxiangyouzhan. Nongxiang 18 had a significant higher average relative content of heptanal than Yuxiangyouzhan. For other volatile compounds, (E)-2-hexenal (Buttery et al.,^1988^; Yang et al., 2008; Goufo et al., 2010 , 2011), 1-hexanol (Mahatheeranont et al., 2001; Goufo et al., 2010, 2011; Mathure et al., 2014), octane (Laohakunjit and Noomhorm, 2004 ), 1-heptanol (Mahatheeranont et al., 2001; Yang et al., 2008; Goufo et al., 2010, 2011; Liyanaarachchi et al., 2014), benzyl alcohol (Wongpornchai et al., 2003; Mathure et al., 2014; Liyanaarachchi et al., 2014), benzeneacetaldehyde (Laohakunjit and Noomhorm, 2004 ), and 3,8-dimethylundecane (Mahatheeranont et al., 2001) were also detected in our study. Shading increased the relative content of (E)-2-hexenal in both varieties. There was no significant difference between shading and non-shading treatments in 1-hexanol, 1-heptanol, and benzeneacetaldehyde. Shading (S2 and S3 for Yuxiangyouzhan, S3 for Nongxiang 18) decreased the relative content of octane, and (S1 for Yuxiangyouzhan, S1 and S3 for Nongxiang 18) significantly increased the relative content of benzyl alcohol. The significant difference in 3,8-dimethylundecane was only found for Nongxiang 18 in S1 and S2 treatments. The results suggest that shading treatments may have a selective effect on the metabolism of the volatile compounds in fragrant rice.

Rice yield, grain quality and 2-AP content are key qualities for aromatic rice. In this study, we have assessed the impact of shading impact on aromatic rice yield, grain quality and 2-AP content, and some related traits. In addition to providing further insight into shading effects on crop yield and quality, and the interaction between 2-AP accumulation and stress in fragrant rice, the results of this study may have implications for rice cultivation in an environment being dramatically influenced by anthropogenic factors. Air pollution can have a shading effect, causing substantial decreases in surface level solar intensity (Jáuregui and Luyando, 1999; Qian et al., 2006), and China is well-known to experience extreme levels of air pollution (Chan and Yao, 2008) including in major agricultural regions such as the Yangtze River delta (Shao et al., 2006). There is evidence that the impact of air pollution has decreased annual solar radiation levels in Guangzhou and even the whole South China region (Liu et al., 2014). Our results suggest that high pollution levels, particularly during grain filling, could influence yield, quality and aroma in elite Chinese fragrant rice varieties. While an increased content of 2-AP as a result of the shading effect of air pollution may be desirable, yield loss and loss of grain quality would clearly be detrimental.

## Conclusions

Here, we have demonstrated that shading during grain filling has significant effects on yield and quality traits in rice, and leads to the accumulation of GABA and 2-AP. These results further emphasize the important relationship between stress and 2-AP production in fragrant rice. Continuing advances in the understanding of this relationship have great potential for the optimization of both aroma production and stress resistance in elite rice varieties.

## Materials and methods

### Plant materials and treatment conditions

Two aromatic rice cultivars, Yuxiangyouzhan and Nongxiang 18 were used in this study. These are the main commercial fragrant rice cultivars in South China. Field experiment was carried out from July to November, 2013 at South China Agricultural University’s Experimental Farm. This region has a humid subtropical monsoonal climate. The field consists of sandy, loamy soil and has been under paddy cultivation for many years. The main soil properties of the experimental site were as follows: pH, 4.88; organic matter content, 25.65%; total N, 1.362%; total P, 0.958%; total K, 17.520%.

The experiment was arranged in split-plot design with varieties as the main plots and shading treatment as the subplots with three duplications. After years of planting, it is well-known that the duration of grain filling in the two varieties employed for this study is approximaltey 30 days. Based on previous studies, we found that a very strong shading level, corresponding to a 90% reduction in full natural light (as measured by a Luxmeter, model ZDS-10, China) resulted in a substantial increase in pest and diseases damage. Therefore, we employed a shading level equivalent to a 67% reduction of full natural light (as measured by a, Luxmeter, model ZDS-10, China) created by one layer of black netting. Four shading treatments were studied: (i) S0: the whole phase during grain filling without shading, taken as the control; (ii) S1: shading during the whole phase of grain filling (30 days, from October 3rd to November 2nd); (iii) S2: shading during the early phase of grain filling (15 days, from October 3rd to October 18th); (iv) S3: shading during the later phase of the grain filling (15 days, from October 18th to November 2nd). The whole grain filling stage was from the end of pollination to grain dry-down, that is from R5 to R9 in the report Counce et al. (2000), equivalent to about 30 days for the two experimental varieties. As outlined above, the whole grain filling stage was devided into two stages, one was the early phase of grain filling (15 days), the other was the later phase of grain filling (15 days).

The area for each subplot was 16 m^2^. Seeds were sown on July 15th and transplanting was performed on August 7th at a density of 20 cm × 20 cm (about 400 hills per plot) with two seedlings per hill. Plants were harvested on November 5th. Fertilizer (1500 kg∙ha^−1^; N, 12.5%; P_2_O_5_, 6.0%; K_2_O, 10.0%; organic matter, 15.0%) was applied 60% at basal, 40% at tillering stage. Irrigation, pest and diseases management, and weed control were the same in all treatments following the guidelines recommended by the province.

### Sampling and measurement

#### Determination of dry weight

Plants from ten randomly selected hills were taken to the laboratory at maturity. Plants were separated into leaves, (stem + sheath) and panicles. Sample were then oven-dried at 80°C to constant weight for the determination of dry weight.

#### Determination of total nitrogen content

Total nitrogen content in grain was determined as described by Lu (1999). Briefly, the dried samples of ground grains (about 0.3 g) were digested using the H_2_SO_4_-HClO_4_ method. The digestion was then used to determine the total nitrogen content by the Kjeldahl method with a 2300 Kjeltec Analyzer Unit (Foss Tecator AB, Sweden).

#### Determination of yield and yield related traits

At maturity, grain yield was measured from one unit sampling area (1 m^2^) within each plot, threshed manually, then sun dried (adjusted to moisture content of ~ 14%). Plants from ten randomly selected hills from each plot were sampled to investigate yield-related traits. Panicle number per m^2^ was measured by counting the panicle numbers of each hill within the unit area (1 m^2^) at three different locations in each plot and the mean value was taken as the final result. Panicles were threshed manually, total number of grains and number of filled grains were counted. Five samples of 1000 grains were taken randomly from filled seeds, weighted to record 1000-grain weight. Harvest index was calculated as the ratio between grain yield and dry weight at maturity, expressed as a percentage.

#### Determination of grain quality

About 1 kg rice grain from each treatment was obtained after storage at room temperature for three months. Brown rice rate was estimated using a rice huller (Jiangsu, China). Milled rice and head rice rates were estimated using a Jingmi testing rice grader (Zhejiang, China). Percentage of grain with chalkiness and chalkiness degree were estimated using an SDE-A light box (Guangzhou, China). Amylose content, protein content, and alkali of grains were determined using an Infratec 1241 grain analyzer (FOSS-TECATOR).

#### Determination of proline content, GABA content and 2-AP concentration

For determination of proline, GABA, and 2-AP, fresh sample of grains from 1 m^2^ area of each plot were harvested and immediately stored at −20°C. Duplicate measurements were taken for each sample and the mean value of the three samples per treatment was taken as the final result.

The proline content was determined according to the method described by Bates et al. (1973). Briefly, grains (about 0.3 g) was homogenized in 5 mL of 3% sulfosalicylic acid, then cooled after heating at boiling water bath for 10 min. Samples filtered and two ml of the filtrate was mixed with 3 mL of ninhydrin reagent ( 1.25 g ninhydrin in 30 mL glacial acetic acid and 20 mL 6 M phosphoric acid) and 2 mL of glacial acetic acid. The reaction mixture was then heated at boiling water bath for 30 min and placed in an ice bath for 20 min before being extracted with 4 ml of toluene. The toluene extraction was then centrifuged at 4000 rpm for 5 min. The absorbance of the red chromophore in the toluene fraction was measured at 520 nm and the amount of proline was determined by comparison with a standard curve and expressed as μg∙g^−1^.

GABA content in grain was determined as described by Zhao et al. (2009) and Yao et al. (2008). Briefly, grain (about 0.5 g) was homogenized in 5 mL of 60% ethanol, treated for 4 hours in a oscillations instrument (HZS-H, China) using a frequency of 200 oscillations per minute. The supernatant was then transferred to a 5 ml centrifuge tube and centrifuged at 8000 rpm for 3 min. 1 mL of supernatant was added to a 10 mL tube, mixed with 0.6 mL 0.2 mol∙L^−1^ (pH 9.0) sodium tetraborate, two mL 5% toluene, and 1 mL 7% sodium hypochlorite, then cooled after heating at 100°C in a water bath for 5 minutes. The absorbance of the reaction solution was measured at 645 nm and the amount of GABA was determined by comparison with a standard curve and expressed as μg∙g^−1^.

Grain samples were evaluated for 2-AP concentration by synchronization distillation and extraction method (SDE) combined with GCMS-QP 2010 Plus (Shimadzu Corporation, Japan) as described by Huang et al. (2012). Briefly, Collidine (2, 4, 6-trimethylpyridine) (Sigma, Switzerland), was used as an internal standard. 10 g of finely ground grain was transferred into a 500 mL round-bottom flask containing 145 mL purified water; 5 mL of 0.914 μg∙mL^−1^ internal standard was then added. A steam distillation continuous extraction head was attached to the flask, and the flask was heated at 150°C by an oil bath pot, ZKYY (Guangzhou, China). Attached to the other head of the steam distillation continuous extraction instrument, diethyl ether (35 mL) was used as the solvent in a 500 mL round-bottom flask, and the flask was heated at 42°C by a water bath pot, HH-2 (Jiangsu, China). During isolation, the steam distillation continuous extraction was maintained at 10°C by a cold water circulation machine, YKKY-LX-300 (Beijing, China). The isolation was performed for 35 min. The ether extract was then dried over sodium sulfate, filtered (0.22 μm filter paper, Shimadzu, Japan), and then directly used to measure 2-AP concentrations with the GCMS-QP 2010 Plus method as described in Huang et al., (2012). The GCMS-QP 2010 Plus working conditions were as followings: gas chromatograph equipped with a Restek Rxi-5 ms (Shimadzu, Japan) silica capillary column (30 m × 0. 32 mm × 0. 25 μm). The auto injector was AOC-20i, SPL1. High purity helium gas (99.999%, Guangzhou Gases Co., LTD, China) was the carrier gas at the flow rate of 2.0 mL∙min^−1^. The temperature of the GC oven was 40°C (1 min), increased at 2°C∙min^−1^ to 65°C and held at 65°C for 1 min, and then increased to 220°C at 10°C∙min^−1^, and held at 220°C for 10 min. The ion source temperature was 200°C. Under these conditions, the retention time of 2-AP was 7.5 min. 2-AP content was expressed as μg∙kg^−1^. The relative content of the aroma compounds were identified on the basis of their mass spectra by comparing the spectra with the records of the NIST library.

### Statistical analysis

Analysis of variance and correlation coefficients were performed using Statistix 8 (Analytical, Tallahassee, Florida, USA). The data were analyzed by one-way analysis of variance to assess differences in yield, yield related traits, total dry weight, harvest index, grain quality, 2-acetyl-1-pyrroline, GABA, proline and total nitrogen between treatments.

## Abbreviations

GABA: γ-aminobutyric acid
2-AP: 2-acetyl-1-pyrroline
GABald: γ-aminobutyraldehyde
PAR: Photosynthetically Active Radiation
SI: solar intensity
